# Pediatrics : The Hygienic and Medical Treatment of Children

**Published:** 1896-06

**Authors:** 


### Book Reviews.

Pediatrics: the Hygienic and Medical Treatment of Children. By Thomas Morgan Rotch, M. D., Professor of the Diseases of Children, Harvard University. Royal 8vo, pp. xii.—1,124. Illustrated. Philadelphia: J. B. Lippincott Company. 1895,

This work is divided into eighteen sections, written in the form of lectures, a style which is hardly an advantage. In the first division the infant at term is discussed, the student being made familiar with its appearance by a beautiful colored plate facing the title-page. Division II. continues with the normal development of the child. The value of the hundred or more pages of these two divisions, which make us acquainted with the normal physiology, anatomy and development of the child, to the young practitioner at least, can hardly be estimated. Comparing these chapters with older works on pediatrics one is impressed with the valuable contribution which Dr. Rotch has made to this branch of medical literature.

The chapter on hygiene of the nursery would make an excellent manual for nursemaids and mothers. This section includes vaccination, illustrated with a fine colored plate. Infant feeding receives the treatment which one would expect from Dr. Rotch;

135 pages are occupied in its consideration. Maternal feeding is considered first. For those beyond the reach of laboratory analyses the author recommends the method of Holt, of New York, for clinical examination of human milk. The necessary instruments are a hydrometer, a pipette and a stoppered 10 c.c. graduated cylinder. But half an ounce of milk is needed. The specific gravity and fat being known, the percentage of proteids may be computed by comparison with the average specific gravity and fat percentage. Sugar is a very constant quantity and the salts are too small in amount to be considered. The careful analyses of milk and the detailed report of cases, under the head of disturbed lactation, are helpful features of this chapter.

Substitute feeding is gone into in much detail, so far as the Walker-Gordon process is concerned, but the physician far beyond the reach of milk laboratories gets but little satisfaction out of the chapter and he may wonder if the cows have not strayed in from an agricultural report.

Division V. considers the premature infant and its management. The blood in infancy and childhood occupies Division VII., one of the most important of the whole work. Much that is new is given here and, as we note throughout the book, the normal conditions are first fully considered. The various blood diseases, primary and secondary, are then taken up and after them follow reports of blood examinations in individual diseases. The author concludes the section with a bibliography.

Diseases of the new-born are fully considered, a bibliography accompanying those rare diseases, acute fatty degeneration and infectious hemoglobinemia. Division IX. deals with diseases of the skin, Division X. deals with syphilis, èrysipelas and the exanthemas. Two colored plates are included in this section. Division XI. takes up the diseases of the nervous system and the myopathies. Nearly 200 pages are occupied with their consideration. The classification of the diseases of the nervous system as given by Dr. Rotch is excellent. He divides them into organic, presumably organic and functional. Functional diseases are further divided into presumably central and reflex. Chorea, epilepsy and insanity are considered under the head of presumably organic. Dr. Rotch acknowledges his indebtedness to Dr. Wm. N. Bullard in the preparation of this section.

Division XII. deals with diseases of the mouth, nose and nasopharynx, including diphtheria. A colored plate illustrates the appearance of the mouth and throat in the various ulcerative and exudative diseases. The antitoxin treatment for diphtheria we would have been glad to see dwelt on at greater length than it here is.

Division XIII. considers the diseases of the gastro-enteric tract. The classification of disease here used is that prepared by Dr. Rotch and Dr. Holt for the American Pediatric Society and adopted by that body. The following section covers diseases of the liver, pancreas, spleen and peritoneum. A relatively small space is devoted to diseases of the genito-urinary organs.

In the lectures devoted to the diseases of the lungs and pleura it is noted that the term capillary bronchitis is eliminated. Diseases of the heart and unclassified diseases, in which section are found rheumatism and rachitis, complete the volume. Throughout the work the illustrations are new and, for the most part, helpful in elucidating the text. In treatment, the author has kept uppermost the idea expressed in the sub-title, "the hygienic and medical treatment of children." This feature commends the book as a guide to the young practitioner, who is ever prone to overdose the baby. The faults of the work are those which any book not encyclopedic in character will possess. It not being possible for one mind to see all the subject in a fair light, some parts seem dwelt upon too lightly. M. J. F.

